# The impact of a pilot continuing professional development module on hospital pharmacists’ preparedness to provide contemporary advice on the clinical use of vancomycin

**DOI:** 10.1186/s40064-016-1966-2

**Published:** 2016-03-15

**Authors:** Cameron J. Phillips, Alice J. Wisdom, Vaughn S. Eaton, Richard J. Woodman, Ross A. McKinnon

**Affiliations:** SA Pharmacy, Flinders Medical Centre, 1 Flinders Drive, Bedford Park, SA 5042 Australia; School of Medicine, Flinders University, Adelaide, SA 5000 Australia; School of Pharmacy and Medical Sciences, University of South Australia, Adelaide, SA 5000 Australia; Flinders Centre for Epidemiology and Biostatistics, Flinders University, Adelaide, SA 5000 Australia; Flinders Centre for Innovation in Cancer, Flinders University, Adelaide, SA 5000 Australia

**Keywords:** Antibiotic, Confidence, Continuing education, Continuing professional development, Knowledge, Vancomycin

## Abstract

**Background:**

Revised international clinical guidelines for the antibiotic vancomycin have changed the advice pharmacists need to provide to medical and nursing colleagues.

**Objectives:**

(1) To determine the self-reported confidence of hospital pharmacists to provide contemporary advice on vancomycin and (2) to evaluate hospital pharmacists’ knowledge to provide contemporary advice on vancomycin following a pilot continuing professional development (CPD) module.

**Methods:**

The study was a prospective two-phase design in an Australian teaching hospital. Phase one: a survey of pharmacist self-reported confidence to eight questions on providing contemporary advice on vancomycin. Responses were recorded using a Likert scales. Phase two: The provision of a pilot online CPD module on vancomycin containing knowledge-based assessment based on a clinical vignette. Likert scales recorded self-reported confidence were reported as median and interquartile range (IQR). Knowledge assessment was reported using descriptive statistics. The main outcome measure were the self-reported confidence, and knowledge of pharmacists regarding provision of contemporary advice on clinical vancomycin use.

**Results:**

Response rates for surveys; confidence n = 35 (72.9 %) and knowledge n = 31 (58.5 %). Phase one: confidence was highest regarding vancomycin dosing and monitoring with 71.4–81.6 % of respondents agreeing or strongly agreeing that they were confident in these domains. Respondents agreeing or strongly agreeing were least confident regarding intravenous administration and infusion related reactions, 57.1 and 45.7 % respectively. Respondents who provided advice on vancomycin >10 times in the prior 12 months reported significantly higher confidence in; therapeutic range 1 (IQR 1–2) versus 2 (IQR 1–3) p = 0.02; amending dosage based on therapeutic drug monitoring results 2 (IQR 1–3) versus 3 (IQR 2–3) p = <0.001, and providing general advice to prescribers on vancomycin 2 (IQR 1–3) versus 2 (IQR 2–4) p = <0.009. Knowledge questions were answered correctly post CPD by >75 % of pharmacists.

**Conclusion:**

Pharmacists’ self-reported confidence to managing vancomycin was variable but generally high. Knowledge scores were consistently high after pharmacists completed a pilot CPD module on vancomycin. These data provides impetus for a randomised controlled study across multiple sites to determine the extent to which pharmacist knowledge on vancomycin can be attributed to completion of an online CPD.

**Electronic supplementary material:**

The online version of this article (doi:10.1186/s40064-016-1966-2) contains supplementary material, which is available to authorized users.

## Background

Confidence and knowledge are important components for healthcare professionals’ ongoing competence to practice. Australian national law requires registered health practitioners to undertake continuing professional development (CPD) with the intention of ensuring knowledge is contemporary (Australian Health Practitioners Regulation Agency 2014). Participating in CPD can meaningfully change knowledge, skills and attitudes of healthcare professionals (Cervero and Gaines 2015). Professional pharmacy organisations in Australian and internationally affirm the importance of maintaining currency of knowledge through CPD (International Pharmaceutical Federation 2002; The Society of Hospital Pharmacists of Australia 2012; Pharmaceutical Society of Australia 2010; Driesen et al. 2007).

Vancomycin is an intravenous antibiotic used for nearly 60 years in the treatment of Gram-positive infections and remains the therapy of choice for methicillin resistant *Staphylococcus aureus* (MRSA) infection (Rybak et al. 2013). While a small number of newer antibiotics to treat MRSA have been licenced by the United States Food and Drug Administration in recent years, it remains vital to reserve these agents for clinical situations when vancomycin fails (Yu et al. 2014). In an era of increasing antibiotic resistance, necessitating higher therapeutic target concentrations and more aggressive dosing of vancomycin (Lomaestro 2011), it is imperative to ensure the ability of pharmacists’ to confidently provide accurate contemporary advice to medical and nursing colleagues. This is important as there have been reported lack of confidence by pharmacists’ post evaluation of programs where pharmacists are required to provide clinical and therapeutic advice, which has led pharmacists to call for more training (Rosenthal et al. 2010).

A North American consensus clinical practice guideline devised by medical and pharmacy experts and revised Australian guidelines on vancomycin have changed the nature of the advice pharmacists provide to medical and nursing staff (Rybak et al. 2009a; Antibiotic Expert Group 2010). Amongst a number of changes in these guidelines, doctors need to frequently prescribe loading doses and larger subsequent doses to achieve a higher serum therapeutic targets and be more cautious with monitoring (Rybak et al. 2009b). Nursing staff are required to infuse vancomycin over revised durations of time to accommodate larger doses (Wilson and Estes 2011; Karch 2012), while being more vigilant in observing for adverse effects, particularly infusion related reactions such as ‘red man syndrome’ (Hoelen et al. 2007).

Since these new recommendations for vancomycin have come into effect, it is unclear how confident pharmacists are in recommending these changes to their professional colleagues. Furthermore, it is uncertain to what extent pharmacists’ confidence in their ability to provide contemporary advice is consistent with their actual knowledge of the revised recommendations for vancomycin.

The aims of this study were threefold. Firstly, to assess pharmacist baseline self-reported confidence in their ability to provide contemporary advice on vancomycin. Secondly, to assess pharmacist knowledge scores after completion of an online vancomycin CPD module. Lastly, to explore any association between pharmacists self-reported confidence scores on providing vancomycin management advice with actual assessed knowledge scores post-completion of a CPD module on vancomycin dosing and monitoring.

## Methods

This pilot study was a prospective two phase design. The study was conducted at Flinders Medical Centre (FMC), a 580-bed university teaching hospital located in Adelaide, South Australia. Study participants were identified from the register of pharmacists employed in the Division of Pharmacy at FMC.

The primary outcomes for the study were (1) to determine if the years of experience as a registered pharmacist have an effect on the self-reported confidence of pharmacists to provide advice on the management of patients receiving vancomycin; and (2) if providing advice on vancomycin more than ten times in the prior 12 months resulted in greater pharmacist self-reported confidence to provide advice on vancomycin management. The secondary outcome was to report pharmacist knowledge scores following a structured online Continuing Professional Development (CPD) module on vancomycin dosing and monitoring.

### Phase one: confidence survey

In June 2012 all identified FMC pharmacists (n = 48) were sent an email inviting them to participate in a survey assessing their confidence in providing vancomycin management advice. The survey was designed to capture self-reported confidence levels in providing advice on effective and safe management of patients receiving vancomycin. Questions were provided on core domains of pharmacists’ involvement in vancomycin management; dosing, therapeutic drug monitoring and intravenous drug administration (see Additional file 1). The survey questions were structured as statements of confidence on the various domains. The degree to which the pharmacists agreed or disagreed with each statement was recorded using a five-point Likert scale (Likert 1932). The following responses represent each point of the Likert scale: Strongly agree (1), agree (2), not sure (3), disagree (4) and strongly disagree (5). The phase one survey was hosted online by Survey Monkey, Portland, OR, USA (www.surveymonkey.net). No incentives were offered to complete the confidence survey.

### Phase two: CPD and knowledge assessment

In February 2013, all identified FMC pharmacists (n = 53) were emailed an invitation to undertake an electronic CPD on Vancomycin Dosing and Monitoring designed by local experts and opinion leaders from FMC. The email contained the CPD with questions (see Additional file 2), a copy of our institutions vancomycin clinical practice guideline, and a link to assessable questions based on a practical clinical vignette which were also hosted on Survey Monkey.

The vancomycin CPD module had formal learning objectives; (1) to familiarise pharmacists with new institutional clinical practice guidelines on vancomycin dosing and monitoring, secondly, (2) to understand the importance of the provision of appropriate and individualised advice on vancomycin in clinical practice to medical and nursing colleagues, (3) to understand how to provide contemporary advice on vancomycin. The CPD module was endorsed by the Society of Hospital Pharmacists of Australia and the Australian Pharmacy Council for accreditation (number S2013/4) for 4 CPD credits. CPD credits accrue toward the Pharmacy Board of Australia’s mandatory requirement for compulsory ongoing professional development. Forty CPD credits are required annually to maintain registration in Australia (Pharmacy Board of Australia 2010). The opportunity to obtain CPD credits was the only incentive offered to undertake the CPD module and complete the assessable questions.

The CPD module contained background on vancomycin regarding; the development of the vancomycin clinical practice guideline, efficacy, safety and reduced bacterial susceptibility to vancomycin along with pharmaceutical formulations available from the state wide pharmacy service that supplies our local health network. Evidence-based, contemporary material was presented to cover all aspects of a pharmacists’ role in providing advice in the management of patients receiving vancomycin. The CPD included a clinical vignette with ten assessable multiple choice questions, with only one correct answer from a choice of four answers. The questions covered similar domains to the confidence survey undertaken in phase one. Supplementary questions were asked of participants’ regarding the relevance of the CPD content and delivery mode (see Additional file 1. The study was granted full ethics approval from the Southern Adelaide Clinical Human Research Ethics Committee (approval number 123.12).

### Statistical analysis and sample size

Data was analysed using the IBM Statistical Package for the Social Sciences (SPSS) version 22.0. Confidence scores recorded in a Likert scale were expressed as a median and IQR, and knowledge was reported using percentage of correctly answered responses for each question. Mann-Whitney U tests were used to compare median confidence scores where applicable with a p value of <0.05 considered statistically significant (Peacock and Peacock 2011). Required sample size was based on 80 % power to detect a mean difference of 0.5 in each Likert scale response between two groups, assuming a standard deviation of 0.55 for each Likert scale response, using a Mann Whitney U test for analysis and an underlying normal distribution of the responses. The means and medians for our data were all similar suggesting a normal distribution and the standard deviations ranged from 0.5 to 1.0 for each question.

## Results

### Phase one: confidence survey

All 48 pharmacists employed in June 2012 in the Division of Pharmacy were sent the confidence survey of which 35 completed (72.9 % response rate). There were 22 (62.9 %) pharmacists with greater than 5 years of practice experience. (Table 1). From the 35 responding pharmacists, 51.4 % reported providing advice on vancomycin to health professionals more than 10 times in the prior 12 months. The majority of respondents agreed or strongly agreed that they possessed confidence in providing advice on vancomycin to health professionals. Median confidence scores ranged from 2 (IQR 1–2) to 3 (IQR 2–3) across the eight questions. Pharmacists’ self-reported confidence was poorest in regards to provision of advice on vancomycin administration rates and the management of infusion related reaction. Pharmacists with less than 5 years of experience were significantly more confident in providing advice on the timing of first vancomycin blood concentrations median score of 1 (IQR 1–3) versus 2 (IQR 1–4) (p = 0.02); and knowing the therapeutic target range 1 (IQR 1–3) versus 2 (IQR 1–3) (p = 0.04) compared to pharmacists with more than 5 years of experience. The years of practice experience had no significant effect on the mean confidence scores for the remaining seven questions (Table 1).

**Table 1:** Hospital pharmacists’ mean self-reported confidence scores providing vancomycin management advice by years of practice experience

Confidence domains	Median (IQR) confidence scores			p value^1^
	All respondents n = 35	<5 years of experience n = 13	>5 years of experience n = 22	
Therapeutic target range	2 (1–2)	1 (1–3)	2 (1–3)	0.04
Timing of first blood concentration	2 (1–2)	1 (1–3)	2 (1–4)	0.02
General advice to prescribers	2 (2–2)	2 (1–3)	2 (1–4)	0.32
Loading dose	2 (2–2)	2 (1–3)	2 (1–4)	0.13
Amending dosing based on TDM	2 (2–3)	2 (1–3)	2 (1–3)	0.10
Frequency of blood concentrations	2 (2–3)	2 (1–4)	2 (1–3)	0.83
Administration rate	2 (2–3)	3 (1–5)	2 (1–4)	0.13
Management of infusion related reactions	3 (2–3)	3 (1–5)	3 (1–5)	0.36

1 =  strongly agree, 2 = agree, 3 = not sure, 4 = disagree, 5 = strongly disagree

*TDM* therapeutic drug monitoring, *IQR* interquartile range, ^1^ Using Mann-Whitney

The confidence of pharmacists with recent experience in providing vancomycin management advice, defined as providing advice greater than ten times in the past 12 months is presented in Table 2. Pharmacists with more recent experience had significantly higher confidence in providing general advice on vancomycin to prescribers, median 2 (IQR 1–3) versus 2 (IQR 2–4) (p = <0.009). These pharmacists also reported a statistically significant greater awareness of the therapeutic target range, median 1 (IQR 1–2) vs 2 (IQR 1-3) (p = 0.02), and confidence to amending vancomycin doses based on sub or supra-therapeutic drug monitoring results compared to pharmacists with less experience, median 2 (IQR 1–3) versus 3 (IQR 2–3) (p = <0.001) (Table 2).

**Table 2:** Hospital pharmacists’ mean self-reported confidence scores on providing vancomycin management advice by recent experience with vancomycin

Confidence domains	Median (IQR) confidence scores		p value^1^
	Provided advice <10 times in last 12 months n = 17	Provided advice >10 times in last 12 months n = 18	
Therapeutic target range	2 (1–3)	1 (1–2)	0.02
Timing of first blood concentration	2 (1–4)	2 (1–3)	0.11
General advice to prescribers	2 (2–4)	2 (1–3)	0.009
Loading dose	2 (1–4)	2 (1–3)	0.10
Amending dosing based on TDM	3 (2–3)	2 (1–3)	<0.001
Frequency of TDM	2 (1–3)	2 (1–4)	0.85
Administration rate	2 (1–4)	2 (1–5)	0.75
Management of infusion related reactions	3 (1–5)	2.5 (1–5)	0.63

1 =  strongly agree, 2 = agree, 3 = neutral, 4 = disagree, 5 = strongly disagree

*TDM* therapeutic drug monitoring, *IQR* interquartile range, ^1^Using Mann-Whitney

### Phase two: CPD and knowledge assessment

In February 2013, all FMC pharmacists (n = 53) were invited to complete a structured CPD module on vancomycin with knowledge assessment. A total of 31 pharmacists (58.5 % response rate) undertook the CPD module and completed the ten assessable questions. There were 17 (54.8 %) pharmacists with greater than 5 years of practice experience. Some 22 (71 %) female participants that completed the questions. Eight of the ten questions elicited correct responses ranging from 93.6–100 %. Questions regarding intravenous administration and management of adverse reactions were answered correctly with a relatively lower frequency of 77.4 % each (Table 3). All respondents agreed or strongly agreed that the CPD activity was of a high educational quality, well presented, up to date, worthwhile and achieved the stated learning objectives. All respondents agreed that the online CPD format suited them.

**Table 3:** Hospital pharmacists’ knowledge scores for domains on providing vancomycin management advice post continuing professional development

Knowledge domain	*n* (%)
Therapeutic target range	31 (100)
Timing to take first blood concentration (in renal impairment)	31 (100)
Frequency of blood concentration following dose stabilisation	30 (96.8)
Timing of blood concentration following dose adjustment	30 (96.8)
Amending dosing based on TDM	30 (96.8)
Subsequent maintenance dose	30 (96.8)
Loading dose	29 (93.6)
Timing of first blood concentration	29 (93.6)
Administration rate	24 (77.4)
Management of infusion related reaction	24 (77.4)

*TDM* therapeutic drug monitoring

### Confidence versus knowledge

**Fig. 1:**
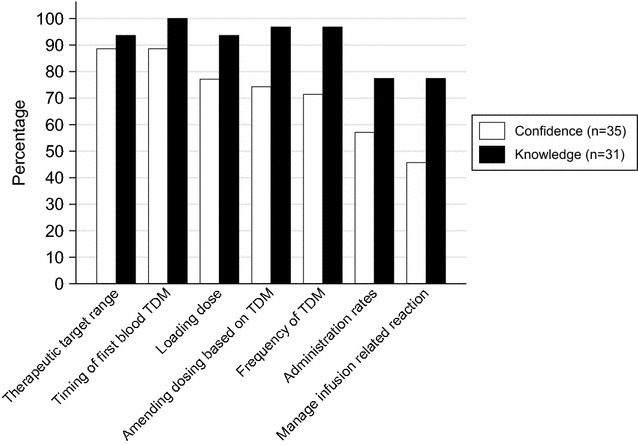
Percentage of hospital pharmacists who agreed or strongly agreed they were confident to provide advice on vancomycin and correct knowledge scores for the same domains post continuing professional development. *TDM* therapeutic drug monitoring

Those pharmacists that self-reported 1 (strongly agree) or 2 (agree) on the Likert scale to questions about confidence in phase one were considered confident. The responses from phase one were plotted against the knowledge scores attained in phase two. Percentage scores were higher for all domains post-completion of CPD as measured by knowledge scores (Fig. 1).

## Discussion

This study sought baseline self-reported confidence of pharmacists to provide contemporary advice on vancomycin clinical management to health professional colleagues in light of revised recommendations. In addition, this study set out to assess knowledge of vancomycin management after completion of a CPD on the topic. There are several strengths to this study. Firstly, the survey questions were designed to capture responses to key domains of vancomycin management reflected in expert consensus guidelines (Rybak et al. 2009a). Secondly, the CPD was developed by local experts and opinion leaders in clinical pharmacy, clinical pharmacology, infectious diseases and clinical education, which is meaningful as a Cochrane review on the effect of local experts on professional practice found that local experts can successfully influence evidence-based practice (Flodgren et al. 2011). Lastly, the questions designed to assess pharmacist knowledge post CPD were derived from a clinical vignette with very practical every-day application to interprofessional advice directly affecting patient care. The use of clinical vignettes in online continuing education has been associated with health professionals being more likely to make evidence-based decisions (Casebeer et al. 2010).

An overall response rate of more than fifty percent of the invited pharmacists was observed in both phases of the study. A number of online surveys of pharmacists related to CPD have attracted lower response rates than those obtained in this current study (Ang et al. 2013; Power et al. 2011, 2008). Specifically the response rate of 73 % obtained in phase one is more than that reported (67 %) by other authors examining pharmacist self-reported confidence (Awaisu et al. 2015). A response rate of 59 % was achieved in phase two which is also greater than that obtained (44 %) from another pharmacist knowledge assessment conducted post CPD in our department (Grzeskowiak et al. 2015). While barriers have been reported to undertaking CPD online (Donyai et al. 2011), this did not seem to be overly problematic for this CPD, with all respondents agreeing the online mode suited them. The only potential incentive to participate was in phase two where CPD credits were available post-completion of the module. The department did not overly encourage pharmacist participation in the study. FMC Pharmacy Department has a view to encourage and support participation in CPD while acknowledging that ultimately it is the responsibility of the individual. Pharmacists must ensure they are competent and capable of discharging their duties as luminaries on pharmacy CPD have recently stated (Tofade et al. 2015). Further, pharmacists should undertake CPD to meet educational needs for their scope of practice (McMahon 2015).

Overall, pharmacists reported greatest self-confidence in the domains of therapeutic drug monitoring, followed by dosing advice and least confident to providing advice on intravenous administration of vancomycin and managing infusion related reactions. Interestingly there was no difference in self-reported confidence for those with less than 5 years’ experience except regarding when to draw the first blood sample to measure a vancomycin concentration. This is noteworthy as it has been reported that if patients have blood drawn too early in the treatment (i.e. serum levels are not at stead-state concentration), this can lead to medical staff misinterpreting concentrations and subsequently prescribing inappropriate dosages (Morrison et al. 2012). What impacted most on pharmacist confidence was recent experience providing advice on vancomycin rather than their years of practice experience. Pharmacists that had provided advice on more than ten occasions in the prior year reported significantly more confidence regarding; provision of general advice on vancomycin to doctors, knowing the therapeutic target range and interpreting concentrations to amend dosage regimens.

After completion of the vancomycin CPD, more than three-quarters of pharmacists surveyed answered all knowledge questions correctly. A score in excess of ninety per cent was obtained for the majority of questions. These results compare similarly to those of other authors where a high knowledge score was attained post completion of a targeted continuing pharmacy education program. (Charpentier et al. 2012).

Questions about managing infusion-related problems and intravenous administration rates generated the lowest self-reported confidence and knowledge scores. These findings are concerning as vancomycin features prominently in medication errors made by nursing staff with intravenous administration errors rating highly (Hoefel et al. 2008; Fahimi et al. 2008). Further, the rate of administration of vancomycin infusion can directly precipitate an infusion related reaction such as red man syndrome (Lilley and Guanci 1995; Garrelts and Peterie 1985; Wallace et al. 1991; Sivagnanam and Deleu 2003; Bauters et al. 2012). This finding suggests more can be done to ensure pharmacist competency in the provision of advice on administration rates and management of infusion-related problems. While baseline confidence on a number of questions was high, a greater knowledge score was attained for all questions post CPD, which is likely to reflect favourably on the content and practical nature of the CPD as was confirmed in responses to supplementary questions.

The results of this study suggest that years of practice as a pharmacist do not routinely translate into higher confidence regarding provision of advice on vancomycin management. Based on these findings, completing a vancomycin CPD module such as the one developed for this study may be of value to pharmacists irrespective of their years of experience if it is clinically relevant to their scope of practice.

### Limitations

Considering potential limitations of this study. The confidence survey in phase one and CPD in phase two were sent to the same departmental email distribution list, however due to workforce issues the absolute number of pharmacists employed varied between the phases of the study. Participants in each phase are thus not necessarily the same individuals. The study was not a before and after design and was conducted in a single centre. However, as more than half the pharmacists employed in our institution participated in each phase of the study, the results are likely to be reflective of the wider cohort. Lastly, selection bias may have been in effect in that those pharmacists who were more confident with contemporary practice or more amendable to improving their knowledge may have chosen to participate, while those in greatest need of updating their knowledge may have elected not to participate.

## Conclusion

Pharmacists provide an important and valuable role assisting their medical and nursing colleague by providing contemporary guidance on medication management. Pharmacists’ ability to provide advice on revised recommendations on vancomycin management is important to ensure the clinically safe and efficacious use of this essential antibiotic. This study adds to the literature on pharmacists’ confidence and knowledge to provide clinical advice. Pharmacists self-reported a variable but generally high degree of confidence in the use of vancomycin. After completion of a pilot online CPD on vancomycin, pharmacists achieved consistently high scores in knowledge assessment. Our results need to be interpreted with caution. A larger and randomised multi-centre study is required to determine if these findings are reproducible.

## Additional files

10.1186/s40064-016-1966-2 Vancomycin Continuing Professional Development with knowledge assessment.
10.1186/s40064-016-1966-2 Questions on pharmacists’ confidence to provide advice on contemporary vancomycin management.

## References

[CR1] Ang HG, Pua YH, Subari NA (2013). Mandatory continuing professional education in pharmacy: the Singapore experience. Int J Clin Pharm.

[CR2] Antibiotic Expert Group (2010). Therapeutic guidelines antibiotic version 14.

[CR3] Australian Health Practitioners Regulation Agency (2014). Code of conduct for registered health practitoners.

[CR4] Awaisu A, Bakdach D, Elajez RH (2015). Hospital pharmacists’ self-evaluation of their competence and confidence in conducting pharmacy practice research. Saudi Pharm J.

[CR5] Bauters T, Claus B, Schelstraete P (2012). Vancomycin-induced red man syndrome in pediatric oncology: Still an issue?. Int J Clin Pharm.

[CR6] Casebeer L, Brown J, Roepke N (2010). Evidence-based choices of physicians: a comparative analysis of physicians participating in Internet CME and non-participants. BMC Med Edu.

[CR7] Cervero RM, Gaines JK (2015). The impact of CME on physician performance and patient health outcomes: an updated synthesis of systematic reviews. J Contin Educ Health Prof.

[CR8] Charpentier MM, Orr KK, Taveira TH (2012). Improving pharmacist knowledge of oral chemotherapy in the community. Ann Pharmacother.

[CR9] Donyai P, Herbert RZ, Denicolo PM (2011). British pharmacy professionals’ beliefs and participation in continuing professional development: a review of the literature. Int J Pharm Pract.

[CR10] Driesen A, Verbeke K, Simoens S (2007). International trends in lifelong learning for pharmacists. Am J Pharm Educ.

[CR11] Fahimi F, Ariapanah P, Faizi M (2008). Errors in preparation and administration of intravenous medications in the intensive care unit of a teaching hospital: an observational study. Aust Crit Care.

[CR12] Flodgren G, Parmelli E, Doumit G et al (2011) Local opinion leaders: effects on professional practice and health care outcomes. Cochrane Database Syst Rev (8):CD000125. doi:10.1002/14651858.CD000125.pub4 10.1002/14651858.CD000125.pub4 PMC4172331 21833939

[CR13] Garrelts J, Peterie J (1985). Vancomycin and the “red man’s syndrome. N Engl J Med.

[CR14] Grzeskowiak LE, Thomas AE, To J (2015). Enhancing continuing education activities using audience response systems: a single-blind controlled trial. J Contin Educ Health Prof.

[CR15] Hoefel HH, Lautert L, Schmitt C (2008). Vancomycin administration: mistakes made by nursing staff. Nurs Stand.

[CR16] Hoelen DWM, Tjan DHT, van Vugt R (2007). Severe local vancomycin induced skin necrosis. Br J Clin Pharmacol.

[CR17] International Pharmaceutical Federation (2002). FIP statement of professional standards continuing professional development.

[CR18] Karch AM (2012). 2013 Lippincott’s nursing drug guide.

[CR19] Likert R (1932). A technique for the measurement of attitudes. Arch Psychol.

[CR20] Lilley LL, Guanci R (1995). Red man syndrome. Am J Nur.

[CR21] Lomaestro BM (2011). Vancomycin dosing and monitoring 2 years after the guidelines. Expert Rev Anti Infect Ther.

[CR22] McMahon GT (2015). Advancing continuing medical education. JAMA.

[CR23] Morrison AP, Melanson SE, Carty MG (2012). What proportion of vancomycin trough levels are drawn too early? Frequency and impact on clinical actions. Am J Clin Pathol.

[CR24] Peacock J, Peacock P (2011). Oxford handbook of medical statistics.

[CR25] Pharmaceutical Society of Australia (2010) The national competency framework for pharmacists in Australia Deakin, Australian Capital Territory

[CR26] Pharmacy Board of Australia (2010). Guidelines on continuing professional development.

[CR27] Power A, Johnson BJ, Diack HL (2008). Scottish pharmacists’ views and attitudes towards continuing professional development. Pharm World Sci.

[CR28] Power A, Grammatiki A, Bates I (2011). Factors affecting the views and attitudes of Scottish pharmacists to continuing professional development. Int J Pharm Pract.

[CR29] Rosenthal M, Austin Z, Tsuyuki RT (2010). Are pharmacists the ultimate barrier to pharmacy practice change?. Can Pharm J.

[CR30] Rybak M, Lomaestro B, Rotschafer JC (2009). Therapeutic monitoring of vancomycin in adult patients: a consensus review of the American Society of Health-System Pharmacists, the Infectious Diseases Society of America, and the Society of Infectious Diseases Pharmacists. Am J Health Syst Pharm.

[CR31] Rybak MJ, Lomaestro BM, Rotschafer JC (2009). Vancomycin therapeutic guidelines: a summary of consensus recommendations from the infectious diseases Society of America, the American Society of Health-System Pharmacists, and the Society of Infectious Diseases Pharmacists. Clin Infect Dis.

[CR32] Rybak MJ, Rotschafer JC, Rodvold KA (2013). Vancomycin: over 50 years later and still a work in progress. Pharmacother.

[CR33] Sivagnanam S, Deleu D (2003). Red man syndrome. Crit Care.

[CR34] The Society of Hospital Pharmacists of Australia (2012). SHPA CODE of ethics.

[CR35] Tofade T, Duggan C, Rouse M (2015). The responsibility of advancing continuing professional development and continuing education globally. Am J Pharm Educ.

[CR36] Wallace MR, Mascola JR, Oldfield EC (1991). Red man syndrome: incidence, etiology, and prophylaxis. J Infect Dis.

[CR37] Wilson J, Estes L (2011). Mayo clinic antimicrobial therapy.

[CR38] Yu T, Stockmann C, Balch AH (2014). Evolution of interventional vancomycin trials in light of new antibiotic development in the USA, 1999–2012. Int J Antimicrob Agents.

