# Supplementary material for: The impact of a pilot continuing professional development module on hospital pharmacists’ preparedness to provide contemporary advice on the clinical use of vancomycin
Source: Springerplus. 2016 Mar 15;5:331. doi: 10.1186/s40064-016-1966-2 (PMC4792834; doi:10.1186/s40064-016-1966-2)
Supplement: Supplementary file 1 — 10.1186/s40064-016-1966-2 Vancomycin Continuing Professional Development with knowledge assessment. [file 40064_2016_1966_MOESM1_ESM.pdf]

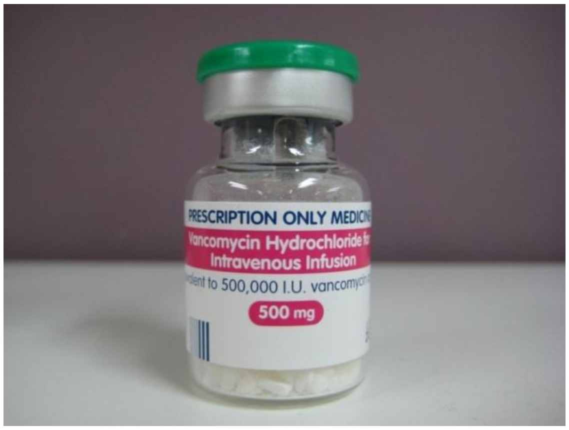

SALHN Vancomycin Dosing & Monitoring Continuing Professional Development

February 2013

Accreditation Number:
S2013/4

SA Pharmacy; FMC, NHS & RGH
Southern Adelaide Local Health Network

Authored by: Cameron Phillips BPharm, MClinPharm, NHMRC TRIP Fellow
Clinical Educator SA Pharmacy, Flinders Medical Centre

SALHN Vancomycin Dosing & Monitoring Continuing Professional Development

Learning objectives

1. To become familiar with the SALHN Vancomycin Dosing & Monitoring Guidelines for Adults.
2. To understand why it is important to appropriately dose and monitor vancomycin
3. To understand how to provide accurate up to date advice on vancomycin dosing & monitoring

Background

The *Vancomycin Dosing and Monitoring Guideline for Adults* has now been approved for use at all sites across the Southern Adelaide Local Health Network (FMC, RGH and NHS). (1) The purpose of this CPD is to provide pharmacists and pharmacy interns with an opportunity to understand some of the reasons for recommendations in the vancomycin guideline and to use the guideline to answer typical questions that may present in the management of patients receiving vancomycin while admitted at the SALHN.

The guideline was developed with input from FMC; Pharmacy, Infectious Diseases & Microbiology, Clinical Pharmacology, Immunology and Hospital in the Home (H@H) after review of the literature and review of Therapeutic guidelines: antibiotic version 14 and the document, Therapeutic monitoring of vancomycin in adult patients: a consensus review of the American Society of Health-System Pharmacists, the Infectious Diseases Society of America, and the Society of Infectious Diseases Pharmacists. (2, 3)

Vancomycin has been in use since the 1950s and yet we are still learning how to use it. (4) Vancomycin use has increased significantly since methicillin (of the penicillin family) resistance to *Staphylococcus aureus* (MRSA) emerged. Vancomycin remains an important antibiotic and is the treatment of choice for serious MRSA infections and for the treatment of infection caused by coagulase-negative staphylococci, in addition vancomycin also has a role in the treatment of infection with *Staphylococcus aureus* when there is a serious allergy to penicillin. (5)

Efficacy, safety & resistance

To use vancomycin safely and with therapeutic effect, appropriate dosing and therapeutic drug monitoring (TDM) are required. As reports of vancomycin treatment failure continue to increase, appropriate dosing and therapeutic drug monitoring is becoming even more important, as vancomycin trough levels <10mg/L are associated with the development of resistant bacteria. (6)

Pharmaceutical products

SA Pharmacy has available 500mg and 1g vials of vancomycin (lyophilised powder for injection).Vancomycin 125mg & 250mg oral capsules are also available for treatment of severe *Clostridium difficile* infection (not the subject of this CPD).

Infectious Diseases (ID) approval

At FMC vancomycin is a restricted antibiotic requiring ID approval and pharmacists should determine if use is approved. The ID approval system permits up to 24 hours supply to be dispensed while awaiting ID approval if the dispensing pharmacist is unable to contact the treating team prior to dispensing or the treating team report they are unable to contact Infectious Diseases to gain ID approval (i.e. weekends, public holidays). Details of the restrictions and exemption for vancomycin use are available in the *Anti-infective reserved protocol.*

Dosing & Administration

Loading dose

In recent years loading doses have demonstrated to safely shorten the time it takes to attain therapeutic target levels. (7, 8, 9) A key US recommendation support a loading dose of 25mg/kg based on actual body weight which is the loading dose chosen for the SALHN Vancomycin Dosing and Monitoring Guideline for Adults. (3) The loading dose has been capped at 2g maximum. A loading dose is not required to be used in all patients however it will be appropriate for a great majority of patients. If a patient has a serious infection then a loading dose is advisable. In any dose calculation round the dose to the nearest 250mg to assist nursing staff with calculations for preparation of the infusion.

Maintenance doses

The first maintenance dose is determined by creatinine clearance (CrCl) and subsequent maintenance doses are determined by TDM and CrCl. For example if a patient has a CrCl >90mL/min, a dose of 1.5g 12 hourly is recommended (except in patients ≤60kg where 25mg/kg is recommended). Maintenance dosing for other CrCl ranges are listed below in Table 1. While some medical staff are keen to use the estimated glomerular filtration rate (eGFR) to choose the dose, remind them that the maximum eGFR reported in Oacis is >60mL/min. With a reported eGFR >60mL/min we are unable to determine which dosing category the patients will be in; i.e. 60-90mL/min or >90mL/min. Always recommend CrCl be calculated, a hyperlink to the eTG CrCl calculator is embedded in the SALHN vancomycin guidelines to work if you are on an SA Health computer.

Table 1

CrCl (mL/min)	Dose
>90	1.5g 12 hourly (if ≤60kg give 25mg/kg 12 hourly)
60-90	1g 12 hourly
20-59	1g 24 hourly
<20	1g & then check vancomycin level at 48 hours

Dosing interval

If the total daily vancomycin dose is >3g, recommend the dosing interval be changed to 8 or 6 hourly. This change in dosing interval will reduce the large fluctuations in vancomycin in vancomycin levels that can occur in patients with high renal clearance i.e. CrCl >90mL/min.

Administration of vancomycin

Vancomycin was originally a very crude formulation referred to as 'Mississippi mud' and hence infusion related reactions such as red man syndrome where reported with more frequency than the current formulation of vancomycin. (10) Today the formulation of vancomycin is a much more purified product and the frequency of infusion reactions appears to be decreased. (5) The SHPA Australian Injectable Drug Handbook recommends that vancomycin should not be infused at a rate greater than 10mg/minute. (11) This recommendation means that a dose of 1g should be administered over 100 minutes and thus a 2g loading dose over 200 minutes, this reference is predominately from the vancomycin Product Information. A number of published practice-based references recommend that 1g of vancomycin can be administered over 60 minutes. (3, 12, 13, 14) This reduced infusion time of 60 minutes for vancomycin 1g is useful for numerous logistical reasons on the ward which is why it is recommend in the SALHN Vancomycin Dosing and Monitoring Guideline for Adults. Pharmacists can elect to continue recommending vancomycin administration over longer periods such as 10mg/min, however if vancomycin is being administered as prophylaxis for surgery please be aware that the infusion should be completed ≤60 minutes prior to skin incision. (14) The duration of vancomycin infusion has recently been reported in an Australia study as being problematic in clinical pathways for patients going to theatre. In this study of 22,549 surgical procedures with 1610 cases receiving vancomycin for surgical prophylaxis for either cardiac or orthopedic surgery, appropriate administration of vancomycin was only recorded in 22% of these cases and much higher rates of surgical site infection with methicillin sensitive *Staphylococcus aureus* were observed with vancomycin when compared with patients receiving a beta-lactam for surgical prophylaxis. (15)

Red man syndrome

Red man-syndrome is a non-immunological reaction which can occur shortly after or during an infusion of vancomycin, which is related to the rate of infusion. The reaction is mediated via histamine release with typical patient presentation of puritis, flushing, erythematous rash (face, neck and upper thorax predominately), fever, chills and in severe cases angioedema and hypotension.

Management of red man syndrome

In the event that you are asked to provide advice regarding the management of red man syndrome the key considerations are;

1. Cease the infusion
2. Recommend an antihistamine such as oral fexofenadine 180mg SR stat
3. Consider adrenaline stat if the patient is hypotension i.e. systolic blood pressure is <90mmHg
4. If the team wishes to continue with vancomycin, recommend the infusion be recommenced at a slower rate i.e. double the original infusion duration that caused the initial reaction and monitor.

<u>Monitoring</u>

Vancomycin levels

Vancomycin levels should always be taken as a trough level i.e. approximately 1 hour pre-dose. (16) Peak levels are no longer considered useful. (10)

What is the Current therapeutic range for vancomycin?
In 2010 SA Pathology increased the therapeutic range to 15-20mg/L for intermittent intravenous infusions which is the range chosen for the SALHN Vancomycin Dosing & Monitoring Guideline for Adults. This is consistent with numerous recommendations, with the therapeutic range being increased due to increasing vancomycin minimum inhibitory concentrations (MIC) of *Staphylococcus aureus* and more treatment failure being reported when vancomycin is use for treatment of MRSA infection.

When to take the first vancomycin level?
This is determined by the half-life of the drug and the renal clearance of the patient. For patients with CrCl ≥60mL/min, taking the level before the fourth dose is recommended. If a patient has a CrCl of 20-59mL/min, taking the first vancomycin level is recommend before the third dose is recommended and if CrCl <20mL/min, the guideline recommends taking the level at 48 hours.

When should subsequent vancomycin levels be measured?
Trough levels should be taken every 48 hours until the patient is within therapeutic range (15-20mg/L) then repeated twice weekly. Serum creatinine should also be measured with the same frequency.

Interpretation of vancomycin levels
Always check the time the last dose was given on the NIMC (not just the time it was originally charted for). Vancomycin levels can be used to inform dosage adjustment.

Dose adjustment
There is a dosage adjustment table in the SALHN Vancomycin Dosing & Monitoring Guideline. When a patient's trough level becomes available it can be matched with the level range specified in the table with their current CrCl to identify if a dosage adjustment is required.

Acknowledgements
Thank you to Vaughn Eaton, Director of Pharmacy FMC & NHS, Professor David Gordon, Dept. Microbiology & Infectious Diseases, Dr. Matt Doogue, Dept. Clinical Pharmacology FMC for their review of this CPD and associated questions. Special thanks to Greg Roberts, Research Pharmacist RGH for incorporating the new vancomycin dosing and monitoring recommendations into the application GFR+ which is available on the SALHN intranet.

References

1. Phillips C, Gordon D, Martinello M. Vancomycin dosing and monitoring guidelines for adults. Southern Adelaide Health Service; 2012
2. Antibiotic Expert Group. Therapeutic guidelines: antibiotic. Version 14. Melbourne: Therapeutic Guidelines Limited; 2010.
3. Rybak M, Lomaestro B, Rotschafer JC, Moellering RC Jr, Craig W, Billeter M, Dalovisio JR, Levine DP. Therapeutic monitoring of vancomycin in adult patients: a consensus review of the North American Society of Health-System Pharmacists, and the Society of Infectious Disease Pharmacists. Am J Health-Sys Pharm 2009; 66:82-98.
4. Stevens D. The role of vancomycin in the treatment paradigm. Clin Inf Dis 2006; 42:S51-7
5. Gyssens IC. Glycopeptides and lipopeptides. In: Grayson ML, editor. Kucers' The Use of Antibiotics, 6th ed. Hodder and Arnold/ASM Press, 2010; 571-600.
6. Howden BP, Davies JK, Johnson PDR, Stinear TP, Grayson ML. Reduced vancomycin susceptibility in *Staphylococcus aureus*, including vancomycin-intermediate and heterogenous vancomycin intermediate strains: resistance mechanisms, laboratory detection, and clinical implications. Clin Microbiol Rev 2010; 23:99-139.
7. Truong J, Levkovich BJ and Padiglione AA. Simple approach to improving vancomycin dosing in intensive care: a standardized loading dose results in earlier therapeutic level. Int Med J 2012; 42: 23-28.
8. Wang JT, Fang CT, Chen YC, Chang SC. Necessity of a loading dose when using vancomycin in critically ill patients. J Antimicrob Chemother 2001; 47:246.
9. Phillips CJ, Doan H, Quinn S, Kirkpatrick CM, Gordon DL, Doogue MP. An educational intervention to improve vancomycin prescribing and monitoring. Int J Antimicrob Agents 2013; http://dx.doi.org/10.1016/j.ijantimicag.2012.11.010
10. Levine DP. Vancomycin: a history. Clin Inf Dis 2006; 42 (suppl 1):S5-S12.
11. Burridge N & Deidun D editors. Society of Hospital Pharmacists of Australia, Australian Injectable Drug Handbook 5th edition, Society of Hospital Pharmacists, Collingwood; 2011
12. Vancomycin UpToDate [internet] version 20.12-C21.3 Waltham, MA USA [cited 10/01/2013]. Available from: http://www.uptodate.com
13. Karch AM. 2013 Lippincott's Nursing Drug Guide. Lippincott Williams & Wilkins, Philadelphia; 2012
14. Engleman R, Shahian D, Shemin R, Guy T, Bratzler D, Edwards F, et al. The Society of Thoracic Surgeons Practice Guideline Series. Antibiotic prophylaxis in cardiac surgery, part 2: antibiotic choice. Ann Thorac Surg 2007;83:1569-76.
15. Bull AL, Worth LJ, Richards MJ. Impact of vancomycin surgical antibiotic prophylaxis on the development of methicillin-sensitive *Staphylococcus aureus* surgical site infection. Report form Australian surveillance data (VICNISS). Ann Surg 2012;256: 1089-1092.
16. Morrison AP, Melanson SEF, Carty MG, Bates DW, Szumita PM, Tanasijevic MJ. What proportion of vancomycin trough levels are drawn too early? Am J Clin Path 2012;137:472-478.

Learning Objectives:

1. To become familiar with the SALHN Vancomycin Dosing & Monitoring Guidelines for Adults.
2. To understand why it is important to appropriately dose and monitor vancomycin
3. To understand how to provide accurate up to date advice on vancomycin dosing and monitoring

Pharmacist competency standards* addressed include:

Standard 4.2 Consider the appropriateness of prescribed medicines
Standard 7.1 Contribute to the therapeutic decision-making
Standard 7.2 Provide ongoing medication management
Standard 7.3 Influence patterns of medicine use

Accreditation:

This CPD has been accredited for **2 hours of Group-2 CPD (or 4 CPD credits)** suitable for inclusion in an individual pharmacist's CPD plan.

Accreditation Number:
S2013/4

Questions

Questions **MUST** be answered in the Survey Monkey link provided in the CPD email to accrue CPD points. Please select the **MOST** appropriate answer. You may consult the SALHN Vancomycin Dosing & Monitoring Guideline for Adults while completing the questions as you would in normal practice.

Q1: What is the main site you work at?
A) FMC
B) NHS
C) RGH

Q2: What is your email address (for notification of results for CPD accreditation)?

CASE

Mr. AB is a 55 year old man with type 2 diabetes who is systemically unwell with fever and suspected sepsis/infection in the Acute Medical Unit. After 24 hours his blood culture result is positive (Gram-positive cocci). At 10:00am the treating team decides to commence vancomycin, ID approval has been granted and the team ask you what loading dose of vancomycin should be charted for Mr. AB?

Mr. AB's is 76kg & 176cm. His serum creatinine is 70 micromoles/L
Current medications: metformin 850mg q12 hourly, gliclazide MR 60mg mane & perindopril 5mg mane.
You will need to calculate his creatinine clearance.

Q3: What loading dose of vancomycin would you recommend?
A) 500mg
B) 1g
C) 1.5g
D) 2g

Q4: The registered nurse caring for Mr. AB asks "over what period of time should this loading dose of vancomycin be administered"? You respond by advising
A) Look it up yourself
B) Over 1 hour
C) Over 1.5 hours
D) Over 2 hours

Q5: What subsequent maintenance dose of vancomycin will you recommend be charted Mr. AB?
A) 500mg 12 hourly
B) 1g daily
C) 1g 12 hourly
D) 1.5g 12 hourly

The medical intern is enthusiastic and wants to write the pathology form for vancomycin TDM when the drug is being charted.

Q6: Before which dose would you recommend the first vancomycin level be taken?
A) Before the second dose
B) Before the third dose
C) Before the fourth dose
D) Before the firth dose

Q7: If Mr. AB had a creatinine clearance of 30mL/min, before which dose would you recommend the initial vancomycin level be taken?

A) Before the first dose
B) Before the second dose
C) Before the third dose
D) Before the sixth dose

Q8: The medical intern asks you what is the therapeutic range for vancomycin (intermittent intravenous infusion) recommended by SA Pathology? Your advice is

A) 5-15mg/L
B) 10-25mg/L
C) 15-20mg/L
D) <20mg/L

Q9: Mr. ABs initial vancomycin trough level is reported as 23.2mg/L. What dosage regimen would you recommend?

A) 1g 12 hourly
B) 1.25g 12 hourly
C) Continue current dose
D) Withhold the next dose

Q10: When should Mr. ABs next vancomycin trough level be checked?

A) 24 hours
B) 48 hours
C) 72 hours
D) Further checking of level is not required

Q11: The RMO asks how often a vancomycin level should be taken once Mr. ABs vancomycin level is in target range (provided his renal function is stable).

A) Every day
B) Twice weekly
C) Once a week
D) Once a fortnight

After one week of therapy (ID have recommended his course be 4 weeks in total for MRSA bacteraemia) Mr. AB develops a red neck during his 0800 infusion of vancomycin (his medications are otherwise unchanged). The registrar thinks it looks like 'red man syndrome'. Mr. ABs blood pressure is 135/80mmHg (consistent with his usual readings). The registrar has discussed Mr. ABs presentation with immunology who advises that the reaction is likely to be red man syndrome and not a true IgE-E mediated drug reaction.

Q12: In addition to recommending an antihistamine what advice will you give to the registrar?

A) Continue the vancomycin infusion at half the previous rate
B) Cease the vancomycin infusion, resume in 3 hours at the previous rate
C) Cease the vancomycin infusion, resume in 3 hours at a slower rate
D) Cease the infusion, and recommend another antibiotic
