# Supplementary material for: The impact of a pilot continuing professional development module on hospital pharmacists’ preparedness to provide contemporary advice on the clinical use of vancomycin
Source: Springerplus. 2016 Mar 15;5:331. doi: 10.1186/s40064-016-1966-2 (PMC4792834; doi:10.1186/s40064-016-1966-2)
Supplement: Supplementary file 2 — 10.1186/s40064-016-1966-2 Questions on pharmacists’ confidence to provide advice on contemporary vancomycin management. [file 40064_2016_1966_MOESM2_ESM.pdf]

Additional file 2

Questions on pharmacists' confidence to provide advice on contemporary vancomycin management

Answer the following questions using 5-point Likert scale 1 strongly agree, 2 agree, 3 not sure, disagree, strongly agree.

1. Do you feel confident in general to provide advice to prescribers on vancomycin?
2. Do you feel confident to advise doctors on the initial dose of vancomycin (considering patient renal function)?
3. Do you feel confident to advise when the first blood concentrations of vancomycin can be taken (to accurately reflect steady-state vancomycin concentrations)?
4. Do you feel confident to advise doctors how often blood concentrations of vancomycin should be taken (once the patient has reached the therapeutic target range)?
5. Are you aware of what the therapeutic target range for vancomycin is?
6. Do you feel confident to interpret high or low vancomycin concentrations to provide advice to doctors on amending the vancomycin dose or interval for a patient?
7. Do you feel confident to provide advice to doctors or nursing staff on how to manage an infusion related reaction to vancomycin (red-man syndrome)?
8. Do you feel confident to advise nursing staff on the administration (rate and concentration) of vancomycin?
